# Evaluation of the anti-angiogenic properties of the new selective α_V_β_3 _integrin antagonist RGDechiHCit

**DOI:** 10.1186/1479-5876-9-7

**Published:** 2011-01-13

**Authors:** Gaetano Santulli, Maria Felicia Basilicata, Mariarosaria De Simone, Carmine Del Giudice, Antonio Anastasio, Daniela Sorriento, Michele Saviano, Annarita Del Gatto, Bruno Trimarco, Carlo Pedone, Laura Zaccaro, Guido Iaccarino

**Affiliations:** 1 Department of Clinical Medicine, Cardiovascular & Immunologic Sciences, "Federico II" University of Naples, Italy; 2 Department of Biological Sciences, "Federico II" University of Naples, Italy; 3 Institute of Crystallography (Consiglio Nazionale delle Ricerche, CNR), Bari, Italy; 4 Institute of Biostructures and Bioimaging (Consiglio Nazionale delle Ricerche, CNR), Naples, Italy

## Abstract

**Background:**

Integrins are heterodimeric receptors that play a critical role in cell-cell and cell-matrix adhesion processes. Among them, α_V_β_3 _integrin, that recognizes the aminoacidic RGD triad, is reported to be involved in angiogenesis, tissue repair and tumor growth. We have recently synthesized a new and selective ligand of α_V_β_3 _receptor, referred to as RGDechiHCit, that contains a cyclic RGD motif and two echistatin moieties.

**Methods:**

The aim of this study is to evaluate *in vitro *and *in vivo *the effects of RGDechiHCit. Therefore, we assessed its properties in cellular (endothelial cells [EC], and vascular smooth muscle cells [VSMC]) and animal models (Wistar Kyoto rats and c57Bl/6 mice) of angiogenesis.

**Results:**

In EC, but not VSMC, RGDechiHCit inhibits intracellular mitogenic signaling and cell proliferation. Furthermore, RGDechiHCit blocks the ability of EC to form tubes on Matrigel. *In vivo*, wound healing is delayed in presence of RGDechiHCit. Similarly, Matrigel plugs demonstrate an antiangiogenic effect of RGDechiHCit.

**Conclusions:**

Our data indicate the importance of RGDechiHCit in the selective inhibition of endothelial α_V_β_3 _integrin *in vitro *and *in vivo*. Such inhibition opens new fields of investigation on the mechanisms of angiogenesis, offering clinical implications for treatment of pathophysiological conditions such as cancer, proliferative retinopathy and inflammatory disease.

## Introduction

Angiogenesis is a complex multistep phenomenon consisting of the sprouting and the growth of new capillary blood vessels starting from the pre-existing ones. It requires the cooperation of several cell types such as endothelial cells (ECs), vascular smooth muscle cells (VSMCs), macrophages, which should be activated, proliferate and migrate to invade the extracellular matrix and cause vascular remodeling [1,2]. The angiogenic process is finely tuned by a precise balance of growth and inhibitory factors and in mammalians it is normally dormant except for some physiological conditions, such as wound healing and ovulation. When this balance is altered, excessive or defective angiogenesis occur and the process becomes pathological. Excessive angiogenesis gives also rise to different dysfunctions, including cancer, eye diseases, rheumatoid arthritis, atherosclerosis, diabetic nephropathy, inflammatory bowel disease, psoriasis, endometriosis, vasculitis, and vascular malformations [3]. Therefore the discovery of angiogenesis inhibitors would contribute to the development of therapeutic treatments for these diseases.

The integrins are cell adhesion receptors that mediate cell-cell and cell-matrix interactions and coordinate signaling allowing a close regulation of physiological phenomena including cellular migration, proliferation and differentiation. In particular, the α_V _integrins, combined with distinct β subunits, participate in the angiogenic process. An extensively studied member of this receptor class is integrin α_V_β_3_, that is strongly overexpressed in activated EC, melanoma, glioblastoma and prostate cancers and in granulation tissue, whereas is not detectable in quiescent blood vessels or in the dermis and epithelium of normal skin [4-6]. This integrin participates in the activation of vascular endothelial growth factor receptor-2 (VEGFR-2), providing a survival signal to the proliferating vascular cells during new vessel growth [7,8] and also seems to be essential in the step of vacuolation and lumen formation [9]. It has been also reported that α_V_β_3 _is under the tight control of VEGF: this integrin is not expressed in quiescent vessels [10], but VEGF induces α_V_β_3 _expression *in vitro *and, interestingly, the VEGF and α_V_β_3 _integrin expression are highly correlated *in vivo *[11,12]. Therefore, α_V_β_3 _should be considered a tumor and activated endothelium marker.

α_V_β_3 _is able of recognizing many proteins of the extracellular matrix, bearing an exposed Arg-Gly-Asp (RGD) tripeptide [5,13,14]. Even if different integrins recognize different proteins containing the RGD triad, many studies have demonstrated that the aminoacids flanking the RGD sequence of high-affinity ligands appear to be critical in modulating their specificity of interaction with integrin complexes [15,16].

Several molecules including peptides containing RGD motif [11] have been recently developed as inhibitors of α_V_β_3 _integrin, in experiments concerning tumor angiogenesis, showing a reduction of functional vessel density associated with retardation of tumor growth and metastasis formation [6,17]. So far, the pentapeptide c(RGDf[NMe]V), also known as cilengitide (*EMD 121974*), is the most active α_v_β_3_/α_v_β_5 _antagonist reported in literature [18,19] and is in phase III clinical trials as antiangiogenic drug for glioblastoma therapy [15]. The development of more selective antiangiogenic molecule would help to minimize the side-effects and increase the therapeutic effectiveness.

We have recently designed and synthesized a novel and selective peptide antagonist, referred to as RGDechiHCit, to visualize α_V_β_3 _receptor on tumour cells [20]. It is a chimeric peptide containing a cyclic RGD motif and two echistatin C-terminal moieties covalently linked by spacer sequence. Cell adhesion assays have shown that RGDechiHCit selectively binds α_V_β_3 _integrin and does not cross-react with α_V_β_5 _and α_IIb_β_3 _integrins [20]. Furthermore, PET and SPECT imaging studies have confirmed that the peptide localizes on α_V_β_3 _expressing tumor cells in xenograft animal model [21]. Since α_V_β_3 _is also a marker of activated endothelium, the main purpose of this study was to evaluate *in vitro *and *in vivo *effects of RGDechiHCit on neovascularization. Thus, we first assessed the *in vitro *peptide properties on bovine aortic ECs, and then *in vivo*, in Wistar Kyoto (WKY) rats and c57BL/6 mice, the ability of this cyclic peptide to inhibit angiogenesis.

## Methods

### Peptides

RGDechiHCit was prepared for the *in vitro *and *in vivo *studies as previously described [20]. To test the biological effects of RGDechiHCit, we synthesized the cyclic pentapeptide c(RGDf[NMe]V), also known as cilengitide or *EMD 121974 *[14,19]. We also investigated RGDechiHCit and c(RGDf[NMe]V) peptides degradation in serum. Both peptides were incubated and the resulting solutions were analyzed by liquid chromatography/mass spectrometry (LC/MS) at different times. 20μL of human serum (Lonza, Basel, Switzerland) were added to 8 μL of a 1 mg/ml solution of either RGDechiHCit or c(RGDf[NMe]V) at 37°C. After 1, 2, 4 and 24h, samples were centrifuged for 1min at 10000g. Solutions were analyzed by LCQ Deca XP Max LC/MS system equipped with a diode-array detector combined with an elctrospray ion source and ion trap mass analyzer (ThermoFinnigan, San Jose, CA, USA), using a Phenomenex C_18 _column (250× 2 mm; 5μm; 300 Ǻ) and a linear gradient of H_2_O (0.1%TFA)/CH_3_CN (0.1%TFA) from 10 to 80% of CH_3_CN (0.1%TFA) in 30 min at flow rate of 200μL/min.

### In vitro studies

*In vitro *studies were performed on cell cultures of ECs or VSMCs, cultured in Dulbecco's modified Eagle's medium (DMEM; Sigma-Aldrich, Milan, Italy) as previously described and validated [22,23]. Cell culture plates were filled with 10 μg/cm^2 ^of human fibronectin (hFN, Millipore^®^, Bedford, MA, USA) as described [24]. All experiments were performed in triplicate with cells between passages 5 and 9.

### Cell proliferation assay

Cell cultures were prepared as previously described [25]. Briefly, cells were seeded at density of 100000 per well in six-well plates, serum starved, pre-incubated at 37°C for 30' with c(RGDf[NMe]V) or RGDechiHCit (10^-6 ^M). Proliferation was induced using hFN (100 μg/ml). Cell number was measured at 3, 6 and 20 h after stimulation as previously described [26,27].

### DNA synthesis

DNA synthesis was assessed as previously described [27]. Briefly, cells were serum-starved for 24 h and then incubated in DMEM with [^3^H]thymidine and 5% FBS. After 3, 6 and 20 h, cells were fixed with trichloracetic acid (0.05%) and dissolved in 1M NaOH. Scintillation liquid was added and [^3^H]thymidine incorporation was assessed as previously described [27].

### VEGF quantification

VEGF production was measured as previously described [26]. Briefly, ECs were seeded at a density of 600000 per well in six well plates, serum starved overnight, seeded with c(RGDf[NMe]V) or RGDechiHCit (10^-6 ^M) and then stimulated with hFN for 6 hours. Cultured medium was collected and VEGF production was revealed by western blot.

### Endothelial Matrigel assay

The formation of network-like structures by ECs on an extracellular matrix (ECM)-like 3D gel consisting of Matrigel^® ^(BD Biosciences, Bedford, MA, USA), was performed as previously described and validated [27,28]. The six-well multidishes were coated with growth factor-reduced Matrigel in according to the manufacturer's instructions. ECs (5×10^4^) were seeded with c(RGDf[NMe]V) or RGDechiHCit (10^-6 ^M), in the absence (negative control) or presence (100 μg/ml) of hFN [24]. Cells were incubated at 37°C for 24h in 1 ml of DMEM. After incubation, ECs underwent differentiation into capillary-like tube structures. Tubule formation was defined as a structure exhibiting a length four times its width [27]. Network formation was observed using an inverted phase-contrast microscope (Zeiss). Representative fields were taken, and the average of the total number of complete tubes formed by cells was counted in 15 random fields by two independent investigators.

### Western blot

Immunoblot analyses were performed as previously described and validated [23,28]. Mouse monoclonal antibodies to extracellular signal regulated kinase (ERK2) and phospho-ERK, anti-rabbit VEGF and actin were from Santa Cruz Biotecnology (Santa Cruz, CA, USA). Levels of VEGF were determined using an antibody raised against VEGF-165 (Santa Cruz Biotechnology) [26]. Experiments were performed in triplicate to ensure reproducibility. Data are presented as arbitrary densitometry units (ADU) after normalization for the total corresponding protein or actin as internal control [24].

### In vivo studies

Wound healing assay was performed on 14-week-old (weight 293 ± 21 g) normotensive WKY male rats (Charles River Laboratories, Calco (LC), Italy; n = 18), and Matrigel plugs experiments were carried out on 16-week-old (weight 33 ± 4 g) c57BL/6 mice (Charles River Laboratories, Milan, Italy; n = 13). All animal procedures were performed in accordance with the *Guide for the Care and Use of Laboratory Animals *published by the National Institutes of Health in the United States (NIH Publication No. 85- 23, revised 1996) and approved by the Ethics Committee for the Use of Animals in Research of "Federico II" University [23].

### Wound Healing

The rats (n = 18) were anesthetized using vaporized isoflurane (4%, Abbott) and maintained by mask ventilation (isoflurane 1.8%)[29]. The dorsum was shaved by applying a depilatory creme (Veet, Reckitt-Benckiser, Milano, Italy) and disinfected with povidone iodine scrub. A 20 mm diameter open wound was excised through the entire thickness of the skin, including the *panniculus carnosus *layer, as described and validated [1,28]. Pluronic gel (30%) containing (10^-6 ^M) c(RGDf[NMe]V) (n = 6), RGDechiHCit (n = 7), or saline (n = 5) was placed daily directly onto open wounds, then covered with a sterile dressing. Two operators blinded to the identity of the sample examined and measured wound areas every day, for 8 days. Direct measurements of wound region were determined by digital planimetry (pixel area), and subsequent analysis was performed using a computer-assisted image analyzer (ImageJ software, version 1.41, National Institutes of Health, Bethesda, MD, USA). Wound healing was quantified as a percentage of the original injury size. Eight days after wounding, rats were euthanized. Wounds did not show sign of infection. The lesion and adiacent normal skin were excised, fixed by immersion in phosphate buffered saline (PBS, 0.01 M, pH 7.2-7.4)/formalin and then embedded in paraffin to be processed for immunohistology, as described [1].

### Matrigel Plugs

Mice (n = 13), anesthetized as described above, were subcutaneously injected midway on the dorsal side, using sterile conditions, with 0.2 ml of Matrigel^® ^basement matrix, pre-mixed with 10^-6^M VEGF and 10^-5^M c(RGDf[NMe]V) (n = 4), 10^-6^M VEGF and 10^-5^M RGDechiHCit (n = 5), or 10^-6^M VEGF alone (n = 4). After seven days, mice were euthanized and the implanted plugs were harvested from underneath the skin, fixed in 10% neutral-buffered formalin solution and then embedded in paraffin. Invading ECs were identified and quantified by analysis of lectin immunostained sections, as described [1,2].

### Histology

All tissues were cut in 5 μm sections and slides were counterstained with a standard mixture of hematoxylin and eosin. For Masson's trichrome staining of collagen fibers, useful to assess the scar tissue formation, slides were stained with Weigert Hematoxylin (Sigma-Aldrich, St. Louis, MO, USA) for 10 minutes, rinsed in PBS (Invitrogen) and then stained with Biebrich scarlet-acid fuchsin (Sigma-Aldrich) for 5 minutes. Slides were rinsed in PBS and stained with phosphomolybdic/phosphotungstic acid solution (Sigma-Aldrich) for 5 minutes then stained with light green (Sigma-Aldrich) for 5 minutes [30]. ECs were identified by lectin immunohistochemical staining (Sigma-Aldrich) [2] and quantitative analysis was performed using digitized representative high resolution photographic images, with a dedicated software (Image Pro Plus; Media Cybernetics, Bethesda, MD, USA) as previously described [28].

### Data presentation and statistical analysis

All data are presented as the mean value ± SEM. Statistical differences were determined by one-way or two-way ANOVA and Bonferroni post hoc testing was performed where applicable. A p value less than 0.05 was considered to be significant. All the statistical analysis and the evaluation of data were performed using GraphPad Prism version 5.01 (GraphPad Software, San Diego, CA, USA).

## Results

### Peptides

**Figure 1:**
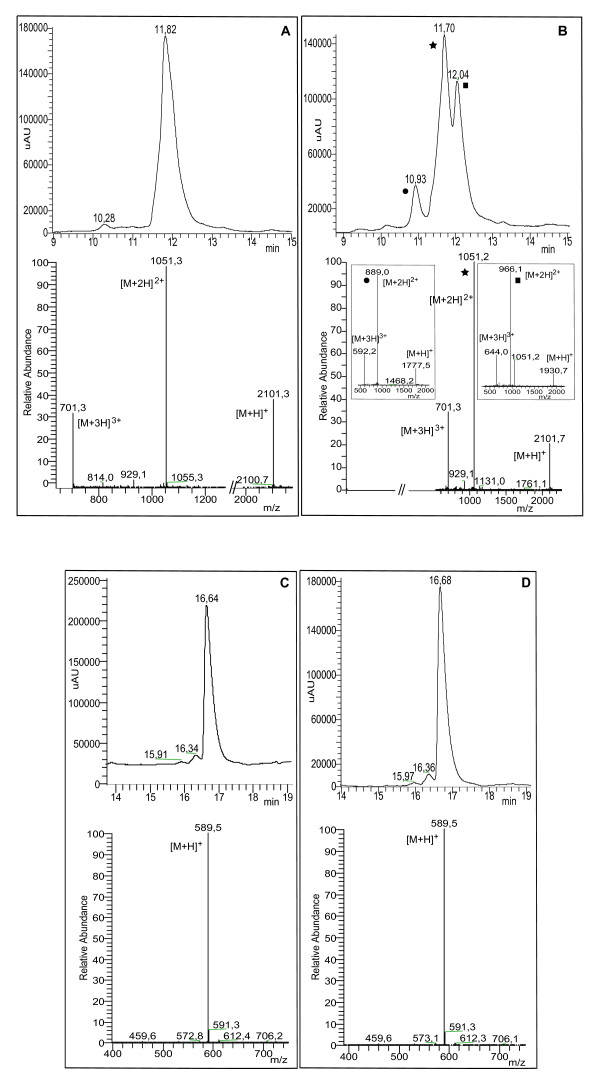
**Reversed-phase high performance liquid chromatography (RP-HPLC) chromatograms and mass spectra at t = 0 and t = 24 h for RGDechiHCit (A and B) and c(RGDf[NMe]V) (C and D), respectively**. In panel B the chromatographic peaks at tr = 11.70 (Black Star), 12.04 (Black Square) and 10.93 min (Black Circle) are marked.

RGDechiHCit and c(RGDf[NMe]V) peptides stabilities were evaluated in serum. The degradation of the peptides were followed by LC/MS. The reversed-phase high performance liquid chromatography (RP-HPLC) of RGDechiHCit before the serum incubation showed a single peak at t_r _= 11.82 min corresponding to the complete sequence (theoretical MW = 2100.1 g mol^-1^) as indicated by the [M+H]^+^, [M+2H]^2+ ^and [M+3H]^+3 ^molecular ion adducts in the MS spectrum (Figure 1A). After 1h, chromatography showed two peaks, ascribable to RGDechiHCit and to a fragment of the complete sequence (theoretical MW = 1929.1 g mol^-1^), respectively, as confirmed by MS spectrum. Finally, after 24h a further peak at t_r _= 10.93 min corresponding to another RGDechiHCit degradation product (theoretical MW = 1775.8 g mol^-1^) appeared, as indicated by the molecular ion adducts in the MS spectrum, although the peaks attributed to the RGDechiHCit and to the first fragment were still present (Figure 1B).

In contrast with RGDechiHCit, c(RGDf[NMe]V) showed high stability in serum. The RP-HPLC profile of the peptide before the incubation showed a single peak at t_r _= 16.64 min, ascribable to the complete sequence by the MS spectrum (Figure 1C). After 24h of incubation chromatogram and mass profiles failed to identify any degradation product (Figure 1D).

Since RGDechiHCit showed a low stability, we replenished antagonists every six hours in experiments involving chronic exposure.

#### In vitro experiments

### Cell proliferation and DNA synthesis

**Figure 2:**
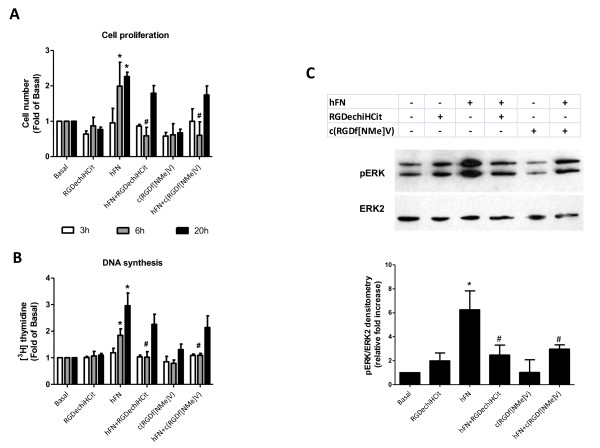
***In vitro *effects of c(RGDf[NMe]V) and RGDechiHCit on cell proliferation (Panel A) and DNA synthesis assessed by [**^**3**^**H]thymidine incorporation **(Panel B) **in bovine aortic endothelial cells (EC). Given alone, c(RGDf[NMe]V) or RGDechiHCit did not affect EC proliferation**. Neverteless, incubation with these α_V_β_3 _integrin antagonists inhibited in a comparable way EC proliferation in response to the mitogenic stimulus, hFN. All experiments depicted in this figure were performed from three to six times in duplicate (* = p < 0.05 vs Basal, # = p < 0.05 vs hFN). **Panel C**. *In vitro *effects of c(RGDf[NMe]V) and RGDechiHCit on EC signal transduction. Extracellular signal regulated kinase (ERK)/mitogen-activated protein kinase activation: western blot of activated (phosphorylated: pERK) ERK2 after hFN-stimulation. Equal amounts of proteins were confirmed via blotting for total ERK. Densitometric analysis (bar graph) showed that hFN stimulation caused ERK activation (* = p < 0.05 vs Basal) and that treatment with α_V_β_3 _antagonists blunted such activation (# = p < 0.05 vs hFN). Error bars show SEM. Representative blots are shown in the inset.

**Figure 3:**
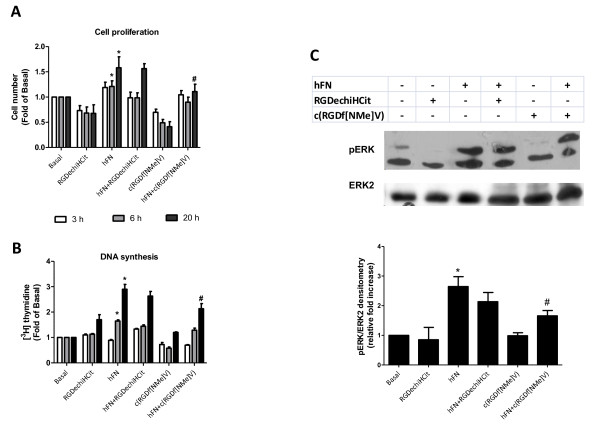
***In vitro *effects of c(RGDf[NME]V) and RGDechiHCit on vascular smooth muscle cell (VSMC) cell proliferation (Panel A) and DNA synthesis assayed by [^3^H]thymidine incorporation (Panel B)**. In this cellular setting, hFN induced a mitogenic stimulus, appreciable especially at 20h. c(RGDf[NMe]V) but not RGDechiHCit at that time-point induced an attenuation of such proliferative response. All experiments were performed from three to five times in triplicate (* = p < 0.05 vs Basal; # = p < 0.05 vs hFN). *In vitro *effects of c(RGDf[NMe]V) and RGDechiHCit on VSMC signal transduction were represented in **Panel C**. Extracellular signal regulated kinase (ERK)/mitogen-activated protein kinase activation: western blot of activated (phosphorylated: pERK) ERK2 after hFN-stimulation. Blots were then stripped and reprobed for either total ERK as a loading control. Densitometric analysis (bar graph) showed that hFN induced ERK phosphorylation (* = p < 0.05 vs Basal) and that treatment with c(RGDf[NMe]V) but not RGDechiHCit decreased such activation (# = p < 0.05 vs hFN). Error bars show SEM. Representative blots are presented in the inset.

Because angiogenesis is intimately associated to EC proliferation, we explored the effects of RGDechiHCit and c(RGDf[NMe]V) on hFN-stimulated EC. In this cellular setting, after 6 hours, both α_v_β_3 _integrin antagonists inhibited in a comparable way the ability of hFN to induce proliferation (hFN: +1.98 ± 0.6; hFN+RGDechiHCit: +0.58 ± 0.24; hFN+c(RGDf[NMe]V): +0.6 ± 0.38 fold over basal; p < 0.05, ANOVA) as depicted in Figure 2A. After 20 hours such inhibitory effect was less marked (Figure 2A). In VSMC there was only a trend of an anti-proliferative effect for these peptides, due to the less evident action of hFN in this specific cellular setting (hFN: +1.21 ± 0.1; hFN+RGDechiHCit: +0.93 ± 0.07; hFN+c(RGDf[NMe]V): +0.9 ± 0.09 fold over basal; NS; Figure 3A).

The effects of RGDechiHCit and c(RGDf[NMe]V) on EC and VSMC proliferation were also measured by assessing the incorporation of [^3^H]Thymidine in response to hFN. This assay confirmed the anti-proliferative action of both these peptides, which is more evident after 6 hours and in ECs (hFN: +1.84 ± 0.24; hFN+RGDechiHCit: + 1.02 ± 0.2; hFN+c(RGDf[NMe]V): + 1.09 ± 0.07 fold over basal; p < 0.05, ANOVA; Figure 2B). On the contrary, the effect of RGDechiHCit on VSMC did not reach statistical significance in comparison to the c(RGDf[NMe]V) used as control (Figure 3B).

### Effects on cellular signal transduction

Since hFN-mediated activation of ERK2 is linked to angiogenesis [16,24,31], we analyzed the ability of RGDechiHCit and c(RGDf[NMe]V) to inhibit hFN-induced phosphorylation of ERK2 in EC and VSMC. In accordance with the results on cell proliferation and [^3^H]Thymidine incorporation, in EC both RGDechiHCit and c(RGDf[NMe]V) significantly inhibited the hFN-induced phosphorylation of mitogen-activated protein ERK2 (Figure 2C). Also, in VSMC, there was no significant inhibition of ERK2 phosphorylation by the RGDechiHCit compund c(RGDf[NMe]V) (Figure 3C).

### Evaluation of VEGF expression

**Figure 4:**
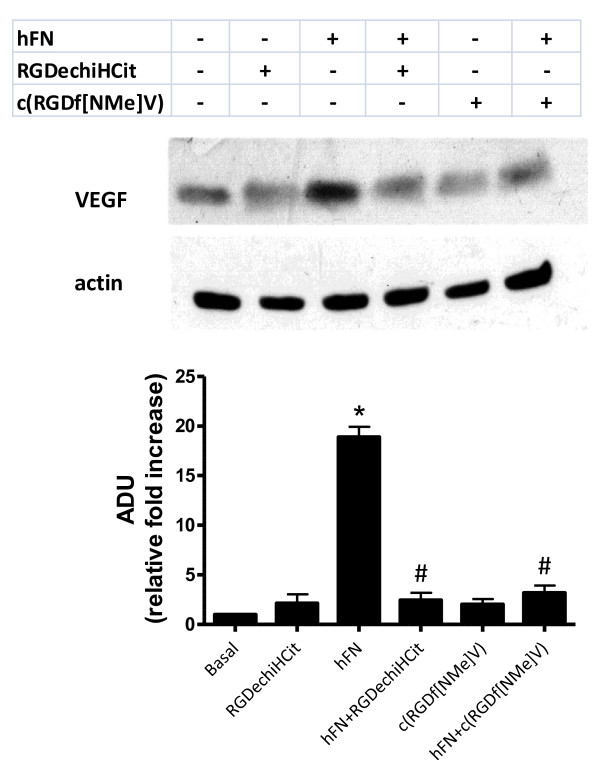
**VEGF production in bovine aortic endothelial cells (ECs) measured by Western blot (inset)**. Shown are VEGF levels after 6 hours of serum starvation. Equal amount of proteins were verified by blotting for actin. Quantification of western blot from all experiments demonstrated that hFN was able to increase VEGF production (* = p < 0.05 vs Basal), while after c(RGDf[NMe]V) or RGDechiHCit treatment VEGF levels returned to basal conditions (# = p < 0.05 vs hFN). All data derived from three different experiments performed in duplicate. The results were expressed as fold increased with respect to the basal condition in untreated samples. Error bars show SEM.

Angiogenesis is largely dependent on ERK2 activation, which in turn promotes cellular proliferation and expression of VEGF. This cytokine promotes infiltration of inflammatory cells, proliferation of ECs and VSMCs and sustains the proangiogenic phenotype [12]. The early release (6 hours) of the cytokine is therefore an important readout when studying angiogenesis *in *vitro. On these grounds, we assessed the expression levels of this pivotal proangiogenetic factor in EC after 6 hours of stimulation with hFN. hFN induces VEGF release and such response was blunted by incubation with either integrin antagonist, as depicted in Figure 4 (hFN: +18.9 ± 1.02; hFN+RGDechiHCit: +2.44 ± 0.76; hFN+c(RGDf[NMe]V): +3.19 ± 0.73 fold over basal, ADU; p < 0.05, ANOVA).

### Endothelial Matrigel assay

The formation of capillary-like tube structures in the ECM by ECs is a pivotal step in angiogenesis and is also involved in cell migration and invasion [26]. To evaluate any potential antiangiogenic activity of our novel integrin antagonist, *in vitro *angiogenesis assays were conducted by evaluating hFN-induced angiogenesis of ECs on Matrigel.

**Figure 5:**
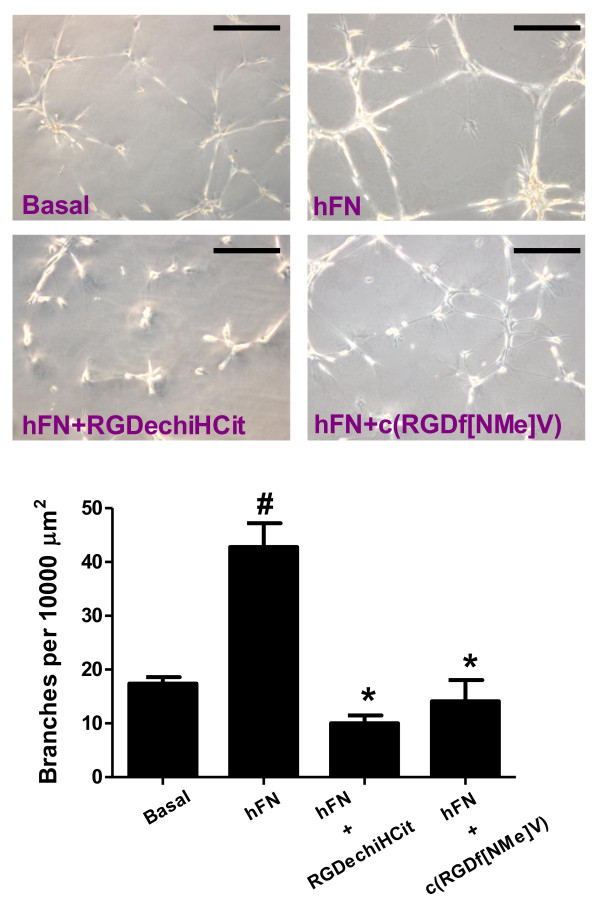
**Representative phase contrast photomicrographs of bovine aortic endothelial cells (ECs) are shown plated on Matrigel**. Both c(RGDf[NMe]V) and RGDechiHCit inhibited hFN-induced tube formation. Microscopy revealed numbers of network projections (branches) formed in each group after 12 h of incubation. Data from three experiments in triplicate are summarized in the graph (* = p < 0.05 vs Basal; # = p < 0.05 vs hFN). Error bars show SEM. The black bar corresponds to 100 μm.

As shown in Figure 5, when ECs were plated on wells coated with Matrigel without the addition of hFN, they showed formation of only a few spontaneous tube structures (17.4 ± 1.2 branches per 10000 μm^2^). On the other hand, when the cells were plated on Matrigel with the addiction of hFN, cells formed a characteristic capillary-like network (42.8 ± 4.4 branches per 10000 μm^2^; p < 0.05 vs Basal, ANOVA). In the presence of RGDechiHCit or c(RGDf[NMe]V), the extent of tube formation hFN-induced was significantly reduced (10.03 ± 1.44; 14.11 ± 3.9, respectively; p < 0.05 vs hFN alone, ANOVA; Figure 5).

#### In vivo experiments

### Wound healing

**Figure 6:**
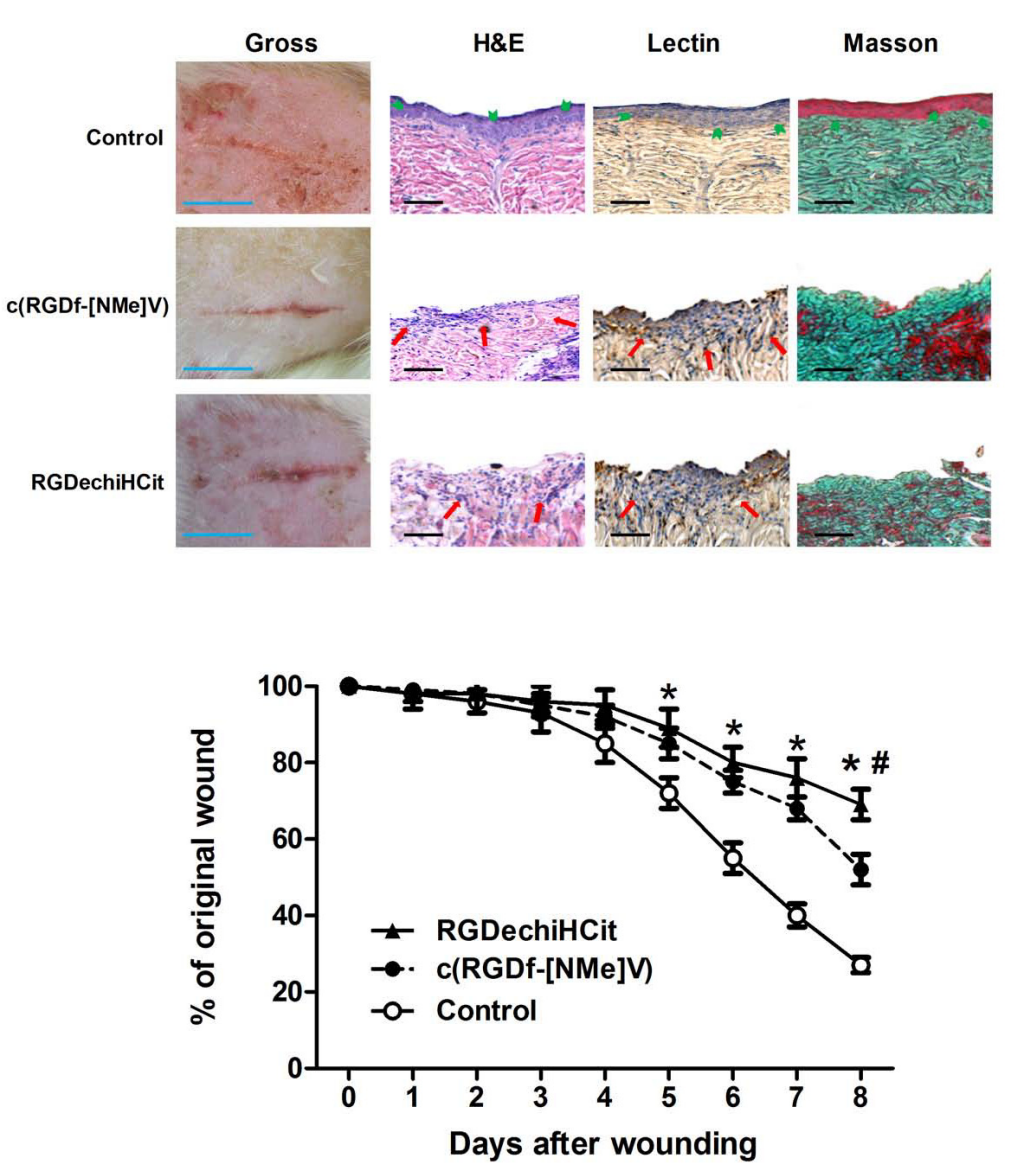
**Both c(RGDf[NMe]V) and RGDechiHCit slowed down the closure of full thickness punch biopsy wounds**. Three to five rats were analyzed at each time point. Gross appearance (representative digital photographs, light blue bar: 1 cm) after 5 days of the wound treated with pluronic gel containing c(RGDf-[NMe]V), RGDechiHCit (10^-6^M) or saline. Diagram of the kinetics of wound closure; * = p < 0.05 vs Control; # = p < 0.05 vs c(RGDf-[NMe]V, ANOVA). Error bars show SEM. Representative sections (5 μm) of wounds excised 8 days after surgery (see Methods): Hematoxylin & Eosin, Lectin immunohistochemistry, Masson's trichrome; black bar: 100 μm. Histological analysis revealed a retarded repair pattern in treated rats, which is consistent with inhibition of angiogenesis in the granulation tissue. In particular, in control animals, epidermal cell growth achieved complete re-epitalization (green arrowheads) and there was a well defined and organized fibrous core of scar tissue. Both in c(RGDf[NMe]V) and RGDechiHCit treated rats there was a chronic inflammatory infiltrate (red arrows) and lectin staining showed (in brown) the presence of vessels in the granulation tissue.

The examination of full-thickness wounds in the back skin showed that both RGDechiHCit and c(RGDf[NMe]V) slowed down healing (Figure 6). At a macroscopic observation, the delay in the wound healing in treated rats was evident, with raised margins, more extensive wound debris and scab, that persisted for at least 7 days after surgery. Moreover, histological analysis showed that while control rats presented a dermal scar tissue consisting of a well defined and organized fibrous core with minimal chronic inflammatory cells, skin wounds exposed to RGDechiHCit or c(RGDf[NMe]V) exhibited a retarded repair pattern. Indeed, there was an intense inflammatory infiltrate, extended from the wound margin into the region of the *panniculus carnosus *muscle and hypodermis. Moreover, the basal epidermis was disorganized and epidermal cell growth failed to achieve re-epithelialization, as shown in Figure 6.

### Matrigel plugs

**Figure 7:**
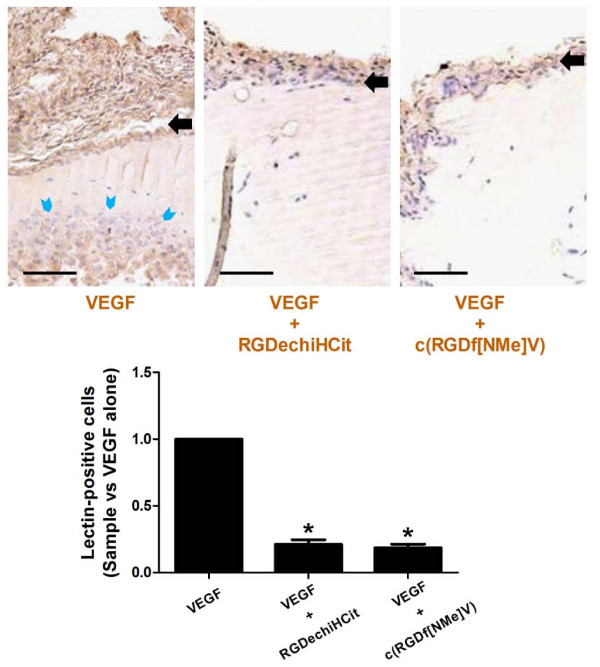
**Representative immunohistochemical sections (5 μm) of subcutaneously injected Matrigel plugs**. ECs were identified (light blue arrowheads) by lectin staining, which gave a brown reaction product, as described in Methods. Both c(RGDf[NMe]V) and RGDechiHCit treatment reduced the number of invading cells from the edge (black arrows) to the core of implanted Matrigel plug. Analysis was conducted in 20 randomly chosen cross-sections per each group. Bar: 400 nm. * = p < 0.05 vs VEGF. Error bars show SEM.

After injection, Matrigel implants containing the angiogenic stimulant VEGF (10^-5 ^M) formed a plug into which ECs can migrate. Matrigel pellets evidenced a significant lower EC infiltration, identified through means of immunohistological lectin staining, in c(RGDf[NMe]V) and RGDechiHCit treated plugs respect to VEGF alone (VEGF+RGDechiHCit: 0.211 ± 0.034; VEGF+c(RGDf[NMe]V): 0.185 ± 0.027 fold over VEGF alone; p < 0.05, ANOVA), as depicted in Figure 7.

## Discussion

In the present study, we evaluated the anti-angiogenic properties of RGDechiHCit peptide *in vitro *on EC and VSMC cells and *in vivo *on animal models of rats and mice. The data here reported recapitulate the well-known antiangiogenic properties of c(RGDf[NMe]V), that was used as control. We previously described the design and synthesis of RGDechiHCit, a novel and selective ligand for α_V_β_3 _integrin, containing a cyclic RGD motif and two echistatin *C*-terminal moieties [20]. *In vitro *studies showed that this molecule is able to selectively bind α_V_β_3 _integrin and not to cross-react with other type of integrins. Furthermore, PET and SPECT imaging studies have confirmed that the peptide localizes on α_V_β_3 _expressing tumor cells in xenograft animal model [21]. Given the presence in the molecule of the RGD sequence it was obvious to speculate that RGDechiHCit acted as an antagonist. Our report is the first evidence that our peptide acts as antagonist for α_V_β_3 _integrin. Its ability to inhibit hFN-induced cell proliferation is comparable to that of c(RGDf[NMe]V), although the half-life is quite reduced.

A major evidence that is brought up by our results is the peculiar selectivity of RGDechiHCit towards EC, as compared to c(RGDf[NMe]V). Indeed, RGDechiHCit fails to inhibit VSMC proliferation *in vitro*, opposite to c(RGDf[NMe]V). We believe that this feature is due to the selectivity of such a novel compound toward α_V_β_3_. Indeed, VSMCs express α_V_β_3 _only during embryogenesis [31], but express other integrins which may be blocked by c(RGDf[NMe]V). On the contrary, α_V_β_3 _is expressed by ECs [8], thus conferring RGDechiHCit selectivity toward this cell type. This issue is relevant cause the effect *in vivo *is similar between the two antagonists on wound healing and Matrigel plugs invasion. Indeed, our data suggest that inhibition of the endothelial integrin system is sufficient to inhibit angiogenesis. It is possible to speculate that the higher specificity of RGDechiHCit for the endothelium would result in a lower occurrence of side effects than the use of less selective inhibitors. This is only an indirect evidence, that needs further investigation in more specific experimental setups. Indeed, of the wide spectrum of integrins that are expressed on the surface of ECs, α_V_β_3 _receptor has been identified as having an especially interesting expression pattern among vascular cells during angiogenesis, vascular remodeling, tumor progression and metastasis [6,32,33]. What is more, two pathways of angiogenesis have been recently identified based on the related but distinct integrins α_V_β_3 _and α_V_β_5 _[4]. In particular, α_V_β_3 _integrin activates VEGF receptors and inhibition of β_3 _subunit has been shown to reduce phosphorylation of VEGF receptors [7], thereby limiting the biological effects of VEGF [1]. Further, Mahabeleshwar and coworkers have shown the intimate interaction occurring between α_V_β_3 _integrin and the VEGFR-2 in primary human EC [12]. The relevance of this molecule to angiogenesis and its potential as a therapeutic target has, therefore, been well established [34,35] and in this report we show that its activity is highly critical for both hFN or VEGF-stimulated ECs proliferation.

Our results concerning RGDechiHCit in angiogenic processes are of immediate translational importance, because deregulation of angiogenesis is involved in several clinical conditions including cancer, ischemic, and inflammatory diseases (atherosclerosis, rheumatoid arthritis, or age-related macular degeneration) [34-36]. Therefore, the research for drugs able to modulate angiogenesis constitutes a crucial investigation field. Since RGDechiHCit is rapidly removed in serum it is possible to increase its effect by engineering the molecule to elongate its lifespan. In the present paper we circumvented this issue by increasing the times of application of the drug both *in vitro *and *in vivo*, or by reducing the times of observation. This issue can be solved by the use of a more stable aromatic pharmacophore that recapitulates the binding properties of RGDechiHCit. Clearly, further investigations are also needed to fully understand the basic cell biological mechanisms underlying growth factor receptors and integrin function during angiogenesis. The knowledge of molecular basis of this complex mechanism remains a challenge of fascinating interest, with clinical implications for treatment of a large number of pathophysiological conditions including but not limited to solid tumors [17,37], diabetic retinopathy [38,39] and inflammatory disease [36].

## Conclusions

The present study indicates the importance of RGDechiHCit in the selective inhibition of endothelial α_V_β_3 _integrin. Such inhibition opens new fields of investigation on the mechanisms of angiogenesis, offering clinical implications for the treatment of several conditions such as proliferative retinopathy, inflammatory disease and cancer.

## Competing interests

We have no financial or personal relationships with other people or organizations that would bias our work. No benefits in any form have been received or will be received from a commercial party related directly or indirectly to the subject of our article.

## Authors' contributions

GS and GI designed research; GS, MFB, MDS, CDG, AA, and DS carried out the experiments; GS and GI performed the statistical analysis; GS, GI and LZ drafted the manuscript; GS, MS, ADG, BT, CP and GI supervised the project; GS and MFB equally contributed to this work. All authors read and approved the final manuscript.
